# Intervention Response to the Trauma-Exposed, Justice-Involved Female Youth: A Narrative Review of Effectiveness in Reducing Recidivism

**DOI:** 10.3390/ijerph17207402

**Published:** 2020-10-12

**Authors:** Ashley Thomann, Latocia Keyes, Amanda Ryan, Genevieve Graaf

**Affiliations:** School of Social Work, University of Texas at Arlington, Arlington, TX 76019, USA; ashley.thomann@mavs.uta.edu (A.T.); amanda.ryan@mavs.uta.edu (A.R.); genevieve.graaf@uta.edu (G.G.)

**Keywords:** trauma exposure, mental health, female justice-involved youth, intervention, reducing recidivism

## Abstract

This study aims to examine current research about trauma-exposed, justice-involved (TEJI) female youth, and evaluate the current literature regarding the effectiveness of gender-specific interventions aimed at reducing their recidivism. Preferred reporting items for systematic reviews and meta-analyses (PRISMA) methodology was utilized to examine quantitative and qualitative literature, published from 2000 through March 2020, about interventions for female justice-involved youth with trauma exposure. Analysis of selected studies utilized an integrated framework based on Andrew’s Risk-Need-Responsivity (RNR) model and Lipsey’s factors of effectiveness, which reviewed studies showing the relationship between female justice-involved youth with mental health symptomologies and trauma. The findings show that effective intervention for this population targets the youth’s negative internal mechanisms related to trauma-subsequent psychosocial problems. These studies suggest that trauma-sensitive modalities have the potential to mitigate the further risk of problematic external behaviors. All studies had limited sample sizes, low follow-up rates, and unknown long-term outcomes. Future research should explore dimensions of sustainability and achieving stability in relation to intervention setting. Selecting the proper venue and facilitator for quality implementation and stability of setting is critical in delivering effective therapies. Modifications in public expectations of juvenile justice policy and practice, from disciplinary to therapeutic approaches, is needed.

## 1. Introduction

Over the past decade, females have become the fastest-growing cohort in the United States juvenile justice system—with young women accounting for a third of juvenile arrests [1]. With only 4% of available programs specifically serving female juveniles and 87% serving all or mostly males, gender-specific interventions and policies are needed to address the growing population of female justice-involved youth [2].

Empirical studies show these detained female adolescents have consistently higher rates of traumatic exposure—with 84–95% having experienced at least one traumatic event [3,4,5]. In fact, many studies illustrate higher reports for trauma in female justice-involved youth than male justice-involved youth [3,6,7,8,9,10,11]. Multiple forms of traumatic stressors have been related to female justice-involved youth. Young women engaged in continual criminality often suffer from the negative consequences of sexual assault or need mental health or drug treatment [11,12,13]. For this reason, justice-involved females’ interpersonal and intrapersonal functionality is significantly compromised. Researchers conducted a meta-analysis to examine the frequency of trauma exposure associated with Post-traumatic stress disorder (PTSD) in males and females for over 25 years. They found that females were two times more likely to meet the criteria for PTSD, and reported higher rates of sexual assault than males [14].

As these youth often face arrest for offenses of interpersonal aggression, experts raise concerns about a disproportionately harsh justice system response to their unique problems [3,15]. Justice system assessments that capture trauma or abuse histories may indicate need for gender-responsive and trauma-informed interventions. Gender-specific and trauma-informed interventions for females are comprehensive and embedded in the experiences of females and avoid trauma-triggering. Such interventions address trauma-response behaviors, and account for and address the psychological effects of violence against women, the structural and economic marginalization of females, and attend to relationships that provide support and meaning for participants [16]. In addition, these interventions may have key elements that enhance self-esteem and ability at the critical stage of female adolescent growth, which permit them to get back on task to achieving developmental milestones [17].

Though it has been over 15 years since attention has been called to the need for trauma-informed and gender-specific programming for females in the justice system, development and empirical evaluation of such interventions has been uncoordinated and sporadic [16]. Though much research has focused on identifying the gender-specific needs of female adults and youth in justice systems, organized assessments of intervention knowledge have focused only on adult women, these studies do not consider dimensions of trauma-informed care, or have evaluated interventions provided to female youth that are not gender-responsive [18,19,20]. To address this gap and to establish a baseline of empirical knowledge, this study aims to review current research about Trauma-Exposed, Justice-Involved (TEJI) female youth, and evaluate the knowledge base about the effectiveness of gender-specific interventions aimed at reducing recidivism in TEJI.

## 2. Background

A majority of research on justice-involved youth relies on research data about the juvenile male population. As such, the female juvenile justice-involved population is an understudied minority within the criminal justice system [3,21]. This is problematic given that females now consist of almost 30% of the juvenile court population.

Detained female adolescents grow up under more adverse living conditions and experience a broader range of persistent mental health and adjustment problems than males—making them particularly vulnerable to social disadvantage and exclusion [3,22]. Female unlawful misconduct also follows specific patterns relating to age and context. Stressors involving family conflict and neighborhoods characterized by poverty, unemployment, and single-parent families are unique risk factors. Comparison of delinquent and non-delinquent females shows that justice-involved females were more likely to be eligible for free or reduced lunch and to have had contact with foster care or child protective services [23].

Recidivism risk also varies by gender. A study completed by the Department of Juvenile Services revealed that of the 427 female youth released from residential facility programming, 58.1% were rearrested, and 18% of girls were reconvicted one year following release [2]. Females with co-morbid substance use and mood disorders are seven times more likely to re-offend than their male counterparts with similar diagnoses. Further, compared with males, female justice-involved youth often display low levels of treatment engagement, are less likely to receive treatment, encounter more mental health obstacles, and are more likely to abandon treatment when compared to male counterparts [23]. While some of this is likely explained by the overall coercive nature of juvenile detention and the problem-oriented, risk management approach to treatment used in these settings, juvenile justice programming suffers from an overall lack of empirically-supported gender-specific programs to address these treatment-related challenges [3].

### 2.1. Gender-Responsive Programming for Justice-Involved Youth

Because gender-specific differences in recidivism risk factors exist among justice-involved youth, the need for empirically supported, gender-specific intervention to reduce recidivism is evident and increasingly necessary, as the population of female justice-involved youth expands. During the last three decades, there has been a growing awareness that change is needed within the criminal justice system with regard to the female justice-involved youth population. With the 1992 reauthorization of the Juvenile Justice and Delinquency Prevention Act (JJDPA), the federal government introduced an amendment that requires all states applying for federal funding to examine their systems for potential gender bias and provide appropriate services for females. This amendment specifies the inclusion of physical health services, mental health services, treatment for trauma/abuse, self-defense, and education [24]. In 1998, The Office of Juvenile Justice and Delinquency Prevention (OJJDP) reissued these recommendations to provide federal funding for more effective, gender-responsive interventions. A few years later, in 2001, the American Bar Association and the National Bar Association published a review of gender bias in the juvenile system calling attention to the need for equity in treatment as it relates to female offenders. Since then, there have been several state actions, including convened committees and task forces, to address gender-specific service needs, policies, and programs based on gender-responsive principles [24].

Researchers report that gender-responsive justice system programming for young women might include counseling, anger management, drug treatment, cognitive-behavioral interventions, sexual abuse treatment, and parenting and life skills classes [25]. Recent studies demonstrate that histories of sexual abuse are more prevalent for justice-involved females than their male counterparts [9,10]. One study revealed 77% of sexual abuse cases were with justice-involved females, contrary to 3% of such history with justice-involved males, proposing an association between trauma and long-lasting criminal misconduct in female youth [26]. Researchers have also illustrated that male and female youth trauma experiences are qualitatively different. For example, justice-involved female youth are likely the victim of violence, such as abuse, while male youth in the justice system learn of violence vicariously, often as a witness [27,28]. Furthermore, a study found females are more likely than males to become involved in the justice system, due to domestic violence perpetrated by youth towards parent(s) [29].

Specifically, girls in the justice system are much more likely to have histories of sexual trauma and are more likely than boys to end up in the system, due to conflict or anger in the home s [29]. These differences suggest that gender-specific interventions need to be targeted at addressing sexual trauma, emotional regulation, and management of interpersonal conflict. Gender-specific programs recognize that romantic relationships and sexuality play a role in their responses in their everyday lives. Thus, teaching girls how to build a “psychologically healthy” relationship, with the focus on their unique developmental needs is vital [25]. The social needs of girls should also be considered, including teaching them how to navigate other girls’ harassment and bullying by identifying positive peer networks, prosocial activities, and choosing romantic partners who are not justice-involved [25,30,31]. Other research suggests that gender-sensitive programming should promote four protective factors for justice-involved girls: The presence of a caring adult, school connectedness, academic success, and spiritual beliefs. Other experts say that gender-responsive principles focus on physical and emotional safety (which aids in counteracting the feelings of fear engendered by past abuse), emphasize healthy, positive relationships to family, and promote self-esteem building to more effectively counteract negative influences [32].

To advance the agenda for developing and disseminating gender-responsive interventions for female youth with justice-involvement, researchers have succeeded in developing and evaluating a modest array of programs and interventions specific to the needs of this population. One female-specific program, Juvenile Justice Anger Management (JJAM), addresses the fact that 54% of girls in a juvenile justice sample report substantial problems with anger and that a significant proportion of female youth offenses are associated with anger and anger-related behaviors [33]. JJAM emphasizes emotion regulation and social problem solving and helps participants manage and counteract hostile attributions [33]. The Good Lives Model (GLM) is another program adaptation for female justice-involved youth, which focuses on meeting girls’ basic human needs and reducing their risk of re-offending [3]. The GLM is ‘strength-based’ in its effort to move beyond the common assessment of risks, deficits, and problems and address capabilities, values, and aspirations [3]. Finally, Functional Family Therapy (FFT) and Multisystemic Therapy (MST) are both empirically supported interventions that are commonly used with justice-involved female youth [34]. One study comparing the effectiveness of these interventions for females concluded that the risks and needs specific to female youth are better addressed by FFT than MST, and a review of justice-system interventions specified that these empirically-supported treatments can be effective for girls, as well as boys [34]. However, this review conceded that few studies directly compared these interventions with ones incorporating gender-specific components or approaches, thus it is unknown if they are equal or superior in outcomes to gender-responsive programs [20].

### 2.2. Trauma Exposure in Justice-Involved Female Youth

Longitudinal research also shows that cumulative trauma early in life is a powerful predictor of justice involvement for both boys and girls. Moreover, studies show that female adolescents in the justice system have consistently higher rates of traumatic exposure than their male counterparts [1,3,35,36,37]. While males are more likely to have witnessed a violent event, females are more likely to have been the victim of violence [28]. One study estimated that up to 60% of girls in a high-security facility had been previously raped or nearly raped, with approximately 60% of those girls having a history of PTSD symptoms and nearly half actively experiencing PTSD symptoms [8,24]. When compared to male counterparts, female justice-involved youth report significantly lower levels of global self-worth and self-esteem within the domains of athletic competence, physical appearance, scholastic competence, and behavioral conduct [3]. These factors make TEJI female youth a particularly vulnerable and challenging population.

Given that history of childhood, sexual abuse trauma is one of the strongest predictors for female juvenile recidivism, gender-specific interventions should also be tailored to address the trauma-exposed population [38]. Emerging research efforts suggest that programming with gender-responsive features is associated with a lower risk of recidivism for girls who present with gender-specific risk factors (e.g., trauma) [2]. One scholar points out that—while gender-responsive principles have positive effects on recidivism for girls who relate to a gender-specific pattern of trauma exposure—effects of the programming on girls who do not demonstrate these gendered risks are deleterious [32].

Because female justice-involved youth are diverse, featuring various pathways into the justice system, effective intervention needs to be guided with the understanding of gender commonalities, as well as gender differences [1]. To appropriately address the needs of the girls in justice settings—particularly those with trauma features—research must determine effective interventions for this population [2].

### 2.3. Principles of Effective Intervention

Within the context of evolving policy, two approaches have been used to evaluate the effectiveness of interventions for female justice-involved youth: Andrew’s Risk-Need-Responsivity (RNR) model and Lipsey’s factors of effectiveness [39,40]. The RNR model consists of three main principles: A risk principle, need principle, and responsivity principle. For the risk principle, this model states that intervention should be matched to the level of an offender’s risk (e.g., longer and more intensive treatment for high-risk offenders and no or minimum treatment for low-risk offenders). Next, the need principle states that dynamic risk factors (i.e., criminogenic needs, the potential risk for ongoing criminality manifested by antisocial peers or substance use) should be the target of treatment because they are changeable and associated with reduced recidivism rates. Last, the responsivity principle states evidence-based treatment should be delivered (specifically, cognitive behavioral interventions), and treatment should correspond to the offender’s characteristics, such as gender, learning style, developmental stage, and level of motivation.

The RNR model is relevant from a risk management perspective because it provides clinicians an effective tool to develop and provide interventions oriented towards solving problems and reducing dynamic risk factors, despite some significant ethical, etiological, and clinical limitations [3]. In a research study on female justice-involved youth applying RNR principles, Welch-Brewer found four distinct groups/profiles with varying levels of risk-needs—Aggression Only (51%), Alcohol and Drug Use (19%), Socioemotional and Family Relationship Problems (24%), and Severe Alcohol and Drug Use (6%)—all warranting a need for varying levels of treatment intensity and different treatment components across subgroups, ranging from less to more extensive [41,42]. These findings show the variation in risk-need profiles across classes indicates heterogeneity within the sample of female offenders, indicating that the needs of these youth may not be fully met using fixed interventions across the board. The study suggested, for example, that trauma-exposed, female justice-involved youth within the Aggression and Drug Use class, may benefit from cognitive behavioral therapy with a relational approach to better respond to their history of trauma and victimization [42].

The Lipsey Factors are another framework for evaluating the effectiveness of interventions, derived from a meta-analysis on all available juvenile justice intervention research from 1958 to 2002. The Lipsey analysis characterizes interventions for justice-involved youth aimed at reducing criminogenic risk factors. Lipsey posits that the most useful guidance for practitioners and the most informative perspective for program developers and researchers will come from the identification of the factors that characterize the most effective programs and the general principles that characterize “what works” to reduce the recidivism of juvenile offenders [40].

Meta-analysis findings suggest that, with other variables statistically controlled, relatively few differences were found in the effectiveness of different types of therapeutic interventions [40]. However, analysis also demonstrates that the higher a youth’s risk, the greater the need for treatment, and the greater impact of that treatment. Further, a well-implemented non-empirically supported intervention, especially when targeted to high-risk offenders, could outperform an evidence-based intervention that is poorly implemented [40,43,44]. The analysis concludes that “it does not take a magic bullet program to impact recidivism, only one that is well made and well-aimed” [40]. The three categories of program characteristics most strongly associated with intervention effects are: (1) The intervention approach and modality (type of treatment), (2) the quantity and quality of treatment provided, and (3) the characteristics of the juveniles receiving that treatment.

Lipsey operationalized intervention approach and modality by identifying the intervention philosophy (surveillance, discipline, deterrence, restorative, counseling, skill building, multiple coordinated services) and treatment modality (e.g., family therapy, individual counseling, social skill building, cognitive-behavioral therapy, academic training, challenge programs, case management, restitution or mediation). Treatment quantity was captured by the total duration of the intervention, as well as how many direct contact hours were involved. He operationalized quality through two proxy-variables: (1) Whether or not implementation problems are reported (e.g., staff turnover or high dropout rates), and (2) whether or not the researcher or developer of the intervention is involved in the delivery of the program—which rests on the assumption that such programs are more carefully implemented and closely monitored than those in routine practice in juvenile justice settings. Program participant characteristics referred to age, gender, race and ethnicity, delinquency risk, history of aggression, and their level of supervision in the justice system.

### 2.4. The Current Study

Because the literature emphasized numerous studies indicating female justice-involved youth report higher levels of trauma than their male counterparts, we focus on gender-responsive interventions to aid female, trauma-exposed youth in recidivism reduction. This study aims to integrate key elements of the RNR and Lipsey’s framework to establish criteria for assessing recidivism-reducing interventions for TEJI adolescent females. This framework will be used for analysis in a narrative review of the peer-reviewed research literature aimed at assessing the state of knowledge about intervention development and effectiveness for TEJI young women.

## 3. Methods

This review was guided by the preferred reporting items for systematic reviews and meta-analyses (PRISMA) method detailed at www.prisma-statement.org to assess quantitative and qualitative literature on effective interventions for female, justice-involved youth with histories of trauma. As the title indicates, PRISMA is an evidence-based minimum set of parameters reporting findings from systematic reviews and meta-analyses. It is often used in evaluating randomized trials but, can also be utilized for reporting systematic reviews of other research and notable evaluations of interventions [45].

### 3.1. Search Methods

Information sources for this search included applicable databases, and the date last searched was 2 March, 2020. The review began with a comprehensive search, at minimum, of the databases: Academic Search Complete, Criminal Justice Database, CINAHL Complete, Criminal Justice Abstracts with Full Text, ERIC, Family Studies Abstracts, MEDLINE, PsycARTICLES, Psychology, and Behavioral Sciences Collection, PsycINFO, and Social Work Abstracts. The author searched titles, abstracts, and subject headings within these databases, and used key search terms to yield the article sample. The search terms included (1) intervention or treatment or therapy or program or strategy, (2) trauma*, (3) female* or girl*, (4) juvenile delinquency or justice-involved youth or youth offenders, and (5) recidivism or re-offending or repeat offenders. Date limits from 2000–2020 were applied, as was a limit for retrieving peer-reviewed articles only.

### 3.2. Study Selection

Articles were selected via adherence to inclusion and exclusion criteria [45]. Due to the study aims to evaluate the effectiveness of gender-responsive interventions for trauma-exposed female adolescents with juvenile justice involvement, the formula excludes any male-inclusive studies, any adult-inclusive studies, any studies not addressing trauma-exposure, and any studies not explicitly evaluating the effectiveness of an intervention. The author extracted and organized all reliable studies to assess the applicability and documented study inclusion decisions.

### 3.3. Data Extraction and Management

For each study, data were extracted for the same set of characteristics: Philosophy and modality of intervention, sample characteristics, study design, intervention duration and quantity, quality in the provision of the intervention, gender-responsive features, outcomes assessed, findings, and study limitations.

### 3.4. Analysis

This review analyzes the resulting sample by describing in the narrative, key features of the included studies, with the aim of critically appraising their qualities, then interpreting and presenting the results [46]. Lipsey’s concepts and Andrew’s Risk-Need-Responsivity (RNR) model were integrated to provide a framework for analysis-oriented toward two objectives: (1) Using descriptive principles to characterize effective programs for trauma-exposed females with justice involvement; and (2) providing a balanced comparative analysis of the effectiveness of the presenting intervention modalities [39,40].

## 4. Results

**Figure 1:**
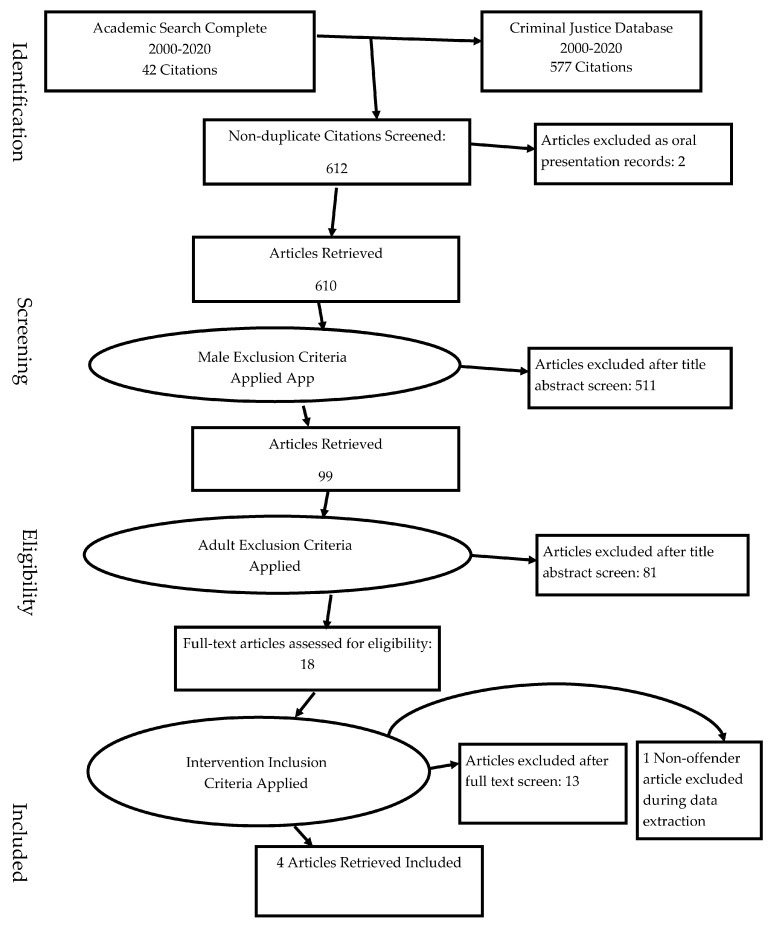
The Roberts-Lewis and colleagues’ study featuring a multi-component substance abuse protocol, (2) the Harold and colleagues’ study using multi-dimensional treatment in foster care (MTFC), (3) the Banks and colleagues explores a modified dialectical behavior therapy (DBT), and (4) the Crosby and colleagues’ study employing trauma-informed teaching as an intervention [47,48,49,50]. The results of the data collected are presented in Table 1: Review of Gender-Responsive, Trauma-Informed Interventions for Justice-Involved Female Youth.

Study selection began with the screening of 619 database-identified articles for duplicates and ineligible records. Seven articles were removed as duplicates, and two additional records were excluded as oral presentation records. Following this screening (see Figure 1. PRISMA Flow Diagram), 610 articles were then assessed for eligibility for inclusion in this review. Initial exclusion examined titles and abstracts for articles employing the term “male, man, men, or boy”. These criteria eliminated 511 articles of the 610 articles leaving 99 articles to review. The 99 articles were then reduced to 18 after the exclusion of non-juvenile oriented articles. The remaining 18 articles were given a full-text screen to assess whether there was an intervention examined in the article, and in so, only five articles were eligible. Finally, after excluding male, adult, and non-interventions, the screen revealed one of the five article interventions did not address female youth with criminal justice involvement, but was instead aimed at examining detention staff. This study was excluded from the final sample. Of the 619 articles, four studies met the eligibility criteria for inclusion in the findings of this narrative review.

### 4.1. Sample

Female youth currently in detention comprised the 30-girl, single sample with no control group within the HEART intervention. It targets females between the ages of 12–18, diagnosed with a substance use disorder, with the aim to change behaviors [47]. Bank and colleagues’ study hosted a sample of 12 girls with 22% participating, due to court mandate. Participants exhibited mental and emotional problems along with symptoms related to self-harm, affective dysregulation, poor interpersonal skill, and/or internalizing or self-destructive behaviors [49]. Crosby and colleagues’ study sample was comprised of 141 female residential placement students with 56% of participants the subject of abuse and neglect petitions, and 44% of participants required by the court, due to delinquency. Participants had high trauma symptomology, lower socio-economic status, as well as the history of neglect and abuse [50]. The Harold and colleagues’ study examined a sample of 166 girls with 81 receiving the MTFC intervention and the other 85 control group receiving group care (GC) service-as-usual. The common sample characteristic was delinquency with mental health impacts caused by maltreatment [48].

### 4.2. Intervention Philosophy

Bank and colleagues’ intervention approach was based on skill building, which included a DBT skills-training group [49]. Crosby and colleagues’ study included two intervention approaches—skill building and counseling—and implemented the Monarch Room (MR) for brief intervention, including talk therapy and problem-solving to help in de-escalation [50]. Roberts-Lewis and colleagues also included skill building and counseling intervention approaches, specifically using group therapy, education, Cognitive behavioral therapy (CBT), 12-step program, education, and other treatments [47]. Harold and colleagues’ intervention approach were based on multiple coordinated services which included treatments, such as support meetings, therapy, 24-h support, and psychiatric consult [48].

### 4.3. Intervention Setting

These studies both occurred in youth detention centers [47,49]. For the school-based intervention, as staff interacted in the classroom, students illustrated appropriate boundaries, conflict resolution, and coping skills to counteract the maladaptive behaviors of traumatized students. This allowed students to learn new ways to socialize and manage stress within the school setting [50]. Harold and colleagues also chose to study an intervention outside the detention environment, a community-based out-of-home care setting [48].

### 4.4. Intervention Type

The Holistic Enrichment for At-Risk Teens (HEART) was employed in one study, a gender-specific substance abuse treatment [47]. Banks and colleagues utilized modified Dialectical Behavior Therapy (DBT) in the form of a DBT skills-training group to aid with self-destructive behaviors, such as suicidality [49]. In the context of a public charter school exclusive to female court-involved students, Crosby and colleagues used trauma-informed teaching intervention to examine school attachment and trauma symptoms [50]. Additionally, researchers examined Multi-dimensional Treatment Foster Care (MTFC) [48].

### 4.5. Intervention Gender-Responsive Components

HEART included several gender-responsive components. Through five treatment stages, participants develop new behaviors at each stage through written goals, activities, and expectations [47]. DBT focused on five main skill areas consisting of an introduction, mindfulness, emotional regulation, interpersonal effectiveness, and distress tolerance [49,51]. The school-based intervention had a two-prong approach using (1) trauma-informed staff training, including Theraplay plus (2) a Monarch Room, which is a non-punitive alternative to traditional discipline, allowing staff to help students de-escalate when emotional states or behaviors interfere with learning [50]. In the community-based intervention, MTFC was utilized as an individualized intervention to improve participant functioning, while including all basic MTFC components [48].

### 4.6. Intervention Quantity

DBT intervention protocol lasted 12 weeks with one 90-min session [49]. Crosby and colleagues’ study occurred over the course of the school-calendar 2013–2014, and participants utilized the Monarch Room any time of day, during a school day, for 10 min or less [50]. With the Harold and colleagues’ study taking place from 1997–2006, with five waves of data, at six-month intervals, average treatment was approximately six months with assessment at five time points over 24 months (baseline, 6, 12, 18, and 24 months) [48]. Roberts-Lewis and colleagues’ study spanned over four and a half years, from 2002–2006, and treatment was available 24-h, 7-days a week [47].

### 4.7. Intervention Quality

With the HEART intervention, strategies focused on the needs of girls and their development with consideration of influences and importance of relationships, power, and disempowering messages sent to females via family, media, and peers. The HEART interventions services were facilitated by the Youth Development Center staff. These services included a therapeutic community modality featuring cognitive behavioral therapy, principles, and strategies of gender-specific services, group psychotherapy, female process groups, psychoeducational groups, psychopharmacotherapy, and 12-step programs [47].

Modifications were made for the traditional DBT format based on the needs of the population. Barriers included feasibility to host evidence-based treatment in the setting, incongruence between values, and interests of setting stakeholders, and maintaining funding. The combination of intervention services included the teaching of DBT skills, references to a published self-help manual, Don’t Let Emotions Run Your Life, and provision of activities based on DBT skills with daily diary cards [49].

The school-based intervention program services included school staff supports plus the utilization of the Monarch Room; specific trauma-informed training, curriculum, attachment-driven, trauma-sensitive teaching strategies, and disciplinary methods. Eight professional development sessions over the course of the year were facilitated by a master’s level social worker and two certified occupational therapists. These trainers provided in-class observation, as well as individual coaching sessions to assist teachers [50].

For the MTFC intervention, experienced program supervisors with small caseloads oversaw the provision of services, while participants had the support of highly trained and supervised homes with state-certified foster parents. The intervention included daily telephone contact with foster parents, weekly group supervision, foster parent support meetings, in-home point-and-level program for girls, and individual therapy for girls. Additional services provided behavioral support, family therapy, monitoring of school responsibilities, and case management of interventions that included 24hr on-call staff support and psychiatric consultation.

### 4.8. Study Design and Outcome Measures

Roberts-Lewis and colleagues’ study had a single-group sample with a multiple repeated measures design, using Pretest-Posttest along with the Problem Oriented Screening Instrument for Teens (POSIT). Participants were not randomly assigned; rather, there was an application and selection process to enter the program, and thus, the study [47]. Banks and colleagues utilized pretest-posttest measures from the Ohio Youth Scales for Problems, Functioning, and Satisfaction—self-report assessment; Problem subscales with internalizing (seven items) and externalizing subscales (nine items); and Becks Depression Inventory-II (BDI-II), another measure for internalizing behavior, also see other assessment examples [49,52]. Crosby and colleagues’ study featured a cross-sectional research design using hierarchical multiple regression. Survey questionnaires comprised of standardized measures were administered by school personnel at the end of the school year [50]. Measurement instruments included the employed Child Report of Post-traumatic Symptoms (CROPS) for trauma, Somers and Gizzi ten-item scale for level of school attachment, and their five-item scale for level of school involvement, and Child and Adolescent Social Support Scale (CASSS) [50,53].

The sample was subject to randomized assignment in the Harold and colleagues’ study. MTFC utilizes Brief Symptom Inventory (BSI) Depression Subscale to screen for psychological problems and measure treatment progress computed as the mean of six items rated on a 5-point Likert-type scale. This instrument examines the trajectory of depressive symptoms in girls with justice involvement referred to out-of-home care who receive MTFC. In addition to testing depression trajectories, they looked at the impact of risk factors like delinquency and childhood abuse on depression and whether they impact intervention effects such that MTFC would benefit those of higher risk. Researchers then used hierarchical linear growth models to assess patterns [48].

### 4.9. Intervention Effectiveness

Participants in Roberts-Lewis and colleagues’ study displayed significant improvement in eight of ten areas of psychosocial functioning. Multiple repeated measures showed significant changes in each of the eight domains. Of the aforementioned, substance use scales indicated 43% of participants with high-risk scores pretest showing a decrease to 23% posttest, and physical health scales showed 43% high-risk scores pretest to 33% posttest. Most notably, 40% had high-risk scores for mental health that decreased to 17% having high-risk scores, and 40% had high-risk scores for family relationships that decreased to 13% posttest. Twenty-seven percent of participants had high-risk scores on the aggressive behavior/delinquency subscale pretest, which then lowered to 17% posttest [47].

DBT treatment proved effective in reducing behavioral and emotional problems commonly experienced by this population. The researchers found decreased internalizing symptoms associated with PTSD, depression, and anxiety. Treatment satisfaction scores and functioning scale scores increased. Problems scale for internalizing behaviors significantly decreased; scores for depression dropped 50%. However, externalizing behaviors saw no significant change [49].

Crosby and colleagues found that students exposed to the school-based intervention who experienced high trauma exposure had unexpectedly elevated school attachment. Participants reported: High levels of trauma symptomology, moderate to high school attachment, involvement, and teacher support, and moderate levels of support from general people, which were all statistically significant. For each unit increase in attachment and social support, trauma symptomology decreased by (0.32) [50].

The MTFC intervention was associated with a 43% reduction in clinical depression relative to the control condition and showed significantly greater rates of deceleration for girls in MTFC versus GC for depressive symptoms. Girls with the highest risk level factors were associated with higher levels of depressive symptoms and benefited more than girls with lower levels of depressive symptoms. The results showed that chronically delinquent girls in MTFC experienced greater decreases in depressive symptoms over a two-year period. Overall, MTFC showed greater benefit for girls with higher levels of initial depressive symptoms [48].

### 4.10. Study Limitations

Random assignment strengthens the Roberts-Lewis and colleagues’ study along with increasing the small sample size. Secondly, the intervention program was voluntary, lending to self-selection bias. Also, the program sample was primarily Caucasian when the larger facility population was African American, leaving generalization to the broader population limited. Finally, the low follow-up rate leaves unknown long-term treatment outcomes [47].

Banks and colleagues’ study was limited by very small sample size, as well as its absence of a control group for comparison measures. Researchers noted the Ohio Scale may not have been best suited, due to participants lacking access to many items. Group plan and structure’s quality assurance was not directly measured, and the study was impacted by dimensions of sustainability and stability of the setting (turnover, politics, funding) [49].

Limitation considerations in Crosby and colleagues’ study noted that research was cross-sectional in nature, and longitudinal research design would be needed to tease out causal relationships. Another limitation includes concerns regarding how other factors may have impacted student perceptions/responses [50].

The final study exhibited Harold and colleagues were presented with several setting-specific limitations. Girls often changed placement following random assignment, and findings might not generalize to girls with more severe symptoms or mood disorder. Other limits include the reliability of measurement based on self-report instrument and the concern that lack of ethnic and racial diversity would not generalize to a more urban setting [48].

## 5. Discussion

All four studies support the correlation of trauma to mental health symptoms in young women with criminal justice involvement, but none actually capture reductions in recidivism as an outcome. The research shows effective intervention for girls with criminal justice involvement should aim to target their negative internal mechanisms related to their trauma-subsequent psychosocial problems. Trauma-sensitive interventions possess the necessary parameters to address the unique needs of these young women. These parameters include targeting trauma-related risk factors with holistic intervention and treatment strategies that address the many, interconnected factors that prevent these young women from developing adaptive social skills, coping strategies, and prosocial behaviors [47]. Findings support the potential for cognitive-behavioral efforts to promote protective factors and inhibit recidivism [47,49]. Other studies have come to similar conclusions regarding cognitive behavior modalities for justice-involved females [30,54].

Outcomes for girls with trauma-related mental health symptoms are often compounded by co-occurring delinquency, with evidence suggesting a closer link between co-occurring delinquency and depression for girls than boys [48]. Substance use disorders among incarcerated girls often appears with other mental health disorders, like depression and anxiety [47,55]. These studies suggest that mitigating internalized trauma symptoms requires employing targeted gender-responsive interventions that improve coping mechanisms. Previous research has also noted coping and management skills aided in gender-responsive programs that greatly reduced recidivism rates for the justice-involved females randomly assigned to either substance recovery or beyond trauma gender-responsive programming. In fact, after one year, they were significantly less likely to be detained again versus the ones who participated in a traditional therapy group [56]. Because female youth with justice-involvement are at risk for depression, poor attachment, poor or damaged self-image, social maladjustment, and anxiety, effective interventions must address these cognitive and emotional patterns [47,48,49,50].

The majority of these studies also link mental health with other important considerations, such as parent-child relationships, parenting, peer association, academics, social support, external behavior, and school attachment [47,49,50]. The Harold and colleagues’ study highlights how context-directed approaches with supports and monitoring may be a particularly critical component of the intervention, due to youths’ limited ability to maintain behavior or thought changes in the context of chaotic, abusive, or resource-limited environments [48]. The trauma from their previous environments—which increases the risk of negative outcomes for court-involved youth—includes higher occurrences of delinquency and recidivism [50,57,58,59]. This delinquency interferes with social development as it causes rejection by social supports and leads to negative mood states [48]. As such, findings suggest the need for symptom-improving interventions to be effective in reducing the odds of future justice-involvement.

### 5.1. Implications for Policy, Practice, and Research

Literature found early age of trauma exposure was substantially correlated with increased post-traumatic stress symptoms in justice-involved females, but not for justice-involved male youth [4]. These findings indicated a need for gender-responsive interventions for female justice-involved youth. Policies that support a trauma-informed justice system should emphasize trauma screening and evaluation, evidence-based trauma management, and foster resilience among youth and families together with engagement [60].

The effectiveness of trauma and gender-sensitive interventions in reducing problematic internalizing and externalizing behaviors that are demonstrated across these four studies suggests the need for modifications in public expectations of juvenile justice policy and practice, from disciplinary to therapeutic. However, along with transformations in public approaches to delinquency, it is important to note that quality implementation and stability of setting can be substantial barriers to delivering effective therapies. Thus, selecting the proper venue and facilitator remains as critical as the content of the intervention itself. Further, calls for change should be tempered by the fact that the evidence base for therapeutic approaches remains limited to demonstrated reductions in risk factors for recidivism—and does not demonstrate direct links to actual reductions in recidivism. Therefore, policies that foster safety and treatment are needed to curb abusive practices that have become commonplace in these institutions.

### 5.2. Policy and Practice Paradigm Shifts

Evidence that gender and trauma-responsive interventions can reduce risk factors for future involvement with the criminal justice system for trauma-exposed young women suggests the need to shift the intervention paradigm from addressing criminal justice problems to meeting criminal justice needs. Adopting approaches that view youth with justice-involvement to be survivors of trauma involves transforming juvenile justice practices from punitive to restorative and evolving detention facilities into treatment sites where girls receive interventions that avert them from further involvement in delinquent behavior [47].

Findings suggest that trauma-responsive programs should be offered in safe, nurturing environments that use culturally competent treatment models, offer dignity and respect to clients, and allow for bonds to be established between treatment staff and the girls involved [16]. Moreover, programs will need to address abandonment, neglect, and abuse histories with an emphasis on building trusting, healthy relationships with intervention facilitators and others [16,61,62]. This environment combats the myriad of resulting psychological problems as experienced by trauma-exposed young women in the justice system [25,63].

### 5.3. Intervention Location and Quality

In addition to being properly targeted to the impact of trauma and associated need to address mental health, analysis reveals interventions are effective to the extent they provide quality implementation and are offered in an optimal setting. Intervention quality, as in the Lipsey review, relates to the involvement of research experts and brings into question the qualifications of those charged with implementing the intervention [40]. With research team involvement and experienced supervisors, interventions may be more effective [48,50]. A quality implementation may also include procedure monitoring, training, education, and experience. The Banks- and colleagues’ study outcome, however, suggests that quality implementation does not necessarily require research involvement or experienced facilitators, rather than highly manualized programming has the potential to offer effective intervention as well [49]. It is also important to note that the quantity of services and duration varied from study to study without apparent impact on effectiveness.

Evaluation of the study characteristics further revealed notable features about the location in which participants received the intervention. In looking at the detention setting studies, the samples were ultimately limited by the setting circumstances. High turnover in both participants and staff fail to offer prime conditions to build stable therapeutic relationships, and effective intervention requires substantial resources and time from participants—which is often limited in detention settings. Given that research shows long-term juvenile incarceration does not decrease re-offending and may actually increase recidivism rates for lower-level youth offenders, time in detention should be as limited as possible for justice-involved youth [64]. As such, study findings suggest that detention may not be an optimal location for the highest impact interventions.

### 5.4. Limitations and Future Research

Beyond limited sample sizes in detention settings, all four studies contended with samples that may not generalize to a broader population. All but one study recognized the unpredictability of participant location changes, while only the Harold and colleagues’ study had a control group for standard comparison. Limited sample sizes, follow-up, and duration are understandable consequences when juvenile detention is not theoretically intended to house offending youth any longer than necessary [48]. Future research should explore dimensions of sustainability and achieving stability in relation to intervention setting. Women with trauma histories are more responsive to trauma-specific interventions than men with trauma histories—which further bolsters the need to expand interventions focused on trauma-exposed female youth, especially justice-involved female youth [65].

Finally, all studies included contended with low follow-up rates with unknown long-term outcomes, suggesting the need for more longitudinal research to identify causal relationships [50]. Further, the majority of outcomes evaluated were primarily risk factors for recidivism (e.g., problematic internal or external behavior; delinquency behavior) and not direct measures of recidivism. As such, claims about societal cost-savings related to appropriate intervention would be inappropriate and unfounded.

## 6. Conclusions

Intervention efforts for trauma-exposed young women with criminal justice involvement require contemplating the presence and gravity of the population’s specific needs. The results from this review suggest that recognition and treatment of trauma-specific mental health needs may be a key component of effective intervention with the potential to impact long-term risk. Interventions that target trauma-specific psychosocial problems via gender-sensitive modalities can help to move justice practices from punitive to restorative, and potentially mitigate the further risk of problematic external behaviors that lead to recidivism. In all, these studies reveal a path forward that maintains notions of justice and gives trauma-exposed young women with criminal-justice involvement the opportunity to constructively restore their lives and decrease future justice involvement.

## Figures and Tables

**Table 1:** Review of Gender-Responsive, Trauma-Informed Interventions for Justice-Involved Female Youth.

Study	Intervention	Location of Intervention	Sample	Design	Duration	Gender-Responsivity	Quality	Quantity	Outcome	Findings	Limitations
[49]	Dialectical behavior therapy (DBT) Skills-training Group	Detention Facility	12 girls; 22% non-voluntary	Pretest/Posttest; Ohio Youth Scales/ Beck Depression Inventory (BDI)-II; Pilot	12 weeks; One 90 min. session per week	Modification based on cognitive and behavioral processes common to girls	Intern facilitators; activities; Highly manualized, no formal training	Dialectical behavior therapy (DBT) skills with Self help manual; Daily diary cards	Impact on internalizing and externalizing behaviors through this modified use of DBT	Internalizing behaviors significantly decreased	Small sample; No control group; Lack stability of setting
[50]	Trauma-informed Teaching	Charter School; exclusive to female youth with court Involvement	141 female students; 56% abuse-neglect petitions 44% court mandate	Cross-sectional with hierarchical regression; Child Report of Post-traumatic Symptoms (CROPS), Child and Adolescent Social Support Scale (CASSS)	2013–2014 School Year	Specific training and curriculum; Attachment driven, trauma-sensitive	Monitored research; trained staff aids	Staff support; Monarch Room	Relationship between school attachment/symptomology	Higher school attachment and lower trauma symptoms	Longitudinal research needed; No address of factors impacting perceptions
[48]	Multi-Dimensional Treatment in Foster Care (MTFC)	Community-based home care	Out-of-166 girls; 81-intervention 85- control	Random assignment; hierarchical linear waves growth model; Brief Symptom Inventory (BSI)	2 years; 5 at 6 month intervals	Context-directed, engaging external supports to overcome internal mechanisms	Experienced supervisors with small caseloads, highly trained homes	Contact with foster parent; Support meetings; Level program; Therapy; 24 h support; Psychiatric consult	Maltreatment History delinquency, and depression levels as moderators of intervention effectiveness	MTFC greater decrease in depression than group care; The more severe the greater decrease	Change in placement following random assignment; Representation may not generalize
[47]	Holistic Enrichment for At-Risk Teens (H.E.A.R.T.)	Detention Facility	30 girls; single group	Pretest (intake)/Posttest (discharge); Problem Oriented Screening Instrument for Teens (POSIT); No random assignment; Pilot	4.5 years	Focus on needs of girls; considers influences of relationships, power, and messages to females	Facility staff, no specific training noted	Cognitive behavioral therapy; Gender specific services; Group Therapy; Education; Pharamaco-therapy; 12-step program	Reduction of psychosocial problems associated with substance abuse and delinquency behaviors	Improved mental health, relationships, education/education/vocation; Lower delinquency risk	Small sample; Lending to self-selection bias; Representation may not generalize

## References

[1] Kerig P.K. (2018). Polyvictimization and girls’ involvement in the juvenile justice system: Investigating gender-differentiated patterns of risk, recidivism, and resilience. J. Interpers. Violence.

[2] Anderson V.R., Walerych B.M., Campbell N.A., Barnes A.R., Davidson W.S., Campbell C.A., Petersen J.L. (2019). Gender-responsive intervention for female justice-involved youth: A quasi-experimental outcome evaluation. Fem. Criminol..

[3] Van Damme L., Fortune C.A., Vandevelde S., Vanderplasschen W. (2017). The good lives model among detained female adolescents. Aggress. Violent Behav..

[4] Dierkhising C.B., Ko S.J., Woods-Jaeger B., Briggs E.C., Lee R., Pynoos R.S. (2013). Trauma histories among justice-involved youth: Findings from the national child traumatic stress network. Eur. J. Psychotraumatol..

[5] Abram K.M., Teplin L.A., Charles D.R., Longworth S.L., McClelland G.M., Dulcan M.K. (2004). Posttraumatic stress disorder and trauma in youth in juvenile detention. Arch. Gen. Psychiatry.

[6] Asscher J.J., van der Put C.E., Stams G.J.J.M. (2015). Gender differences in the impact of abuse and neglect victimization on adolescent offending behavior. J. Fam. Violence.

[7] Brosky B.A., Lally S.J. (2004). Prevalence of trauma, PTSD, and dissociation in court-referred adolescents. J. Interpers. Violence.

[8] Cauffman E., Feldman S., Waterman J., Steiner H. (1998). Posttraumatic stress disorder among female justice-involved youth. J. Am. Acad. Child. Adolesc. Psychiatry.

[9] Foy D.W., Ritchie I.K., Conway A.H. (2012). Trauma exposure, posttraumatic stress, and comorbidities in female adolescent offenders: Findings and implications from recent studies. Eur. J. Psychotraumatol..

[10] Mueser K.T., Taub J. (2008). Trauma and PTSD among adolescents with severe emotional disorders involved in multiple service systems. Psychiatr. Serv..

[11] Tossone K., Wheeler M., Butcher F., Kretschmar J. (2018). The role of sexual abuse in trauma symptoms, delinquent and suicidal behaviors, and criminal justice outcomes among females in a juvenile justice diversion program. Violence Against Women.

[12] Cruise K.R., Ford J.D. (2011). Trauma exposure and PTSD in justice-involved youth. Child. Youth Care Forum.

[13] Simkins S., Katz S. (2002). Criminalizing abused girls. Violence Against Women.

[14] Tolin D.F., Foa E.B. (2006). Sex differences in trauma and posttraumatic stress disorder: A quantitative review of 25 years of research. Psychol. Bull..

[15] Van Damme L., Hoeve M., Vermeiren R., Vanderplasschen W., Colins O. (2016). Quality of life in relation to future mental health problems and offending: Testing the good lives model among detained girls. Law Hum. Behav..

[16] Bloom B., Owens B., Covington S. Gender-Responsive Strategies: Research, Practice, and Guiding Principles for Women Offenders. http://www.nicic:pubs/2003/018017.pdf.

[17] Goodkind S. (2005). Gender-specific services in the juvenile justice system: A critical Examination. Affilia.

[18] Kerig P., Schindler S. (2013). Engendering the evidence base: A critical review of the conceptual and empirical foundations of gender-responsive interventions for girls’ delinquency. Laws.

[19] Gobeil R., Blanchette K., Stewart L. (2016). A meta-analytic review of correctional interventions for women offenders: Gender-neutral versus gender-informed approaches. Crim. Justice Behav..

[20] Leve L.D., Chamberlain P., Kim H.K. (2015). Risks, outcomes, and evidence-based interventions for girls in the US juvenile justice system. Clin. Child. Fam. Psychol. Rev..

[21] Sheahan F. Neglected Needs: Girls in the Criminal Justice System. https://www.penalreform:resource/neglected-girls-criminal-justice-system/.

[22] Giaconia R.M., Reinherz H.Z., Silverman A.B., Pakiz B., Frost A.K., Cohen E. (1995). Traumas and posttraumatic stress disorder in a community population of older adolescents. J. Am. Acad. Child. Adolesc. Psychiatry.

[23] Barrett D., Ju S., Katsiyannis A., Zhang D. (2015). Females in the juvenile justice system: Influences on delinquency and recidivism. J. Child. Fam. Stud..

[24] Walker S.C., Muno A., Sullivan-Colglazier C. (2015). Principles in practice: A multistate study of gender-responsive reforms in the juvenile justice system. Crime Delinq..

[25] Garcia C.A., Lane J. (2013). What a girl wants, what a girl needs: Findings from a gender specific focus group study. Crime Delinq..

[26] Sherman F. (2013). Detention Reform and Girls: Challenges and Solutions.

[27] Sedlak A., McPherson K. (2010). Youth’s Needs and Services.

[28] Espinosa E.M., Sorensen J.R., Lopez M.A. (2013). Youth pathways to placement: The influence of gender, mental health need and trauma on confinement in the juvenile justice system. J. Youth Adolesc..

[29] Gavazzi S.M., Yarcheck C.M., Chesney-Lind M. (2006). Global risk indicators and the role of gender in a juvenile detention sample. Crim. Justice Behav..

[30] Wright E.M., Van Voorhis P., Salisbury E.J., Bauman A. (2012). Gender-responsive lessons learned and policy implications for women in prison: A review. Crim. Justice Behav..

[31] Van Voorhis P., Wright E.M., Salisbury E., Bauman A. (2010). Women’s risk factors and their contributions to existing risk/needs assessment: The current status of a gender-responsive supplement. Crim. Justice Behav..

[32] Day J.C., Zahn M.A., Tichavsky L.P. (2015). What works for whom? The effects of gender responsive programming on girls and boys in secure detention. J. Res. Crime Delinq..

[33] Goldstein N.E.S., Serico J.M., Riggs Romaine C.L., Zelechoski A.D., Kalbeitzer R., Kemp K., Lane C. (2013). Development of the juvenile justice anger management treatment for girls. Cogn. Behav. Pract..

[34] Baglivio M.T., Jackowski K., Greenwald M.A., Wolff K.T. (2014). Comparison of multisystemic therapy and functional family therapy effectiveness: A multiyear statewide propensity score matching analysis of justice-involved youth. Crim. Justice Behav..

[35] Horan J.M., Widom C.S. (2014). Cumulative childhood risk and adult functioning in abused and neglected children grown up. Dev. Psychopathol..

[36] Kerig P.K., Becker S.P., Morizot J., Kazemian L. (2015). Early abuse and neglect as risk factors for the development of criminal and antisocial behavior. The Development of Criminal and Antisocial Behavior.

[37] Malvaso C.G., Delfabbro P., Day A. (2016). Risk factors that influence the maltreatment offending association: A systematic review of the methodological features of prospective and longitudinal studies. Aggress. Violent Behav..

[38] Behnken M.P., Bort A., Borbon M. (2017). Race and gender recidivism differences among juvenile mental health court graduates. Juv. Fam. Court J..

[39] Andrews D.A., Dvoskin J.A., Skeem J.L., Novaco R.W., Douglas K.S. (2012). The risk-need-responsivity (RNR) model of correctional assessment and treatment. American Psychology–Law Society Series. Using Social Science to Reduce Violent Offending.

[40] Lipsey M.W. (2009). The primary factors that characterize effective interventions with justice involved youth: A meta-analytic overview. Vict. Offenders.

[41] Collins L.M., Murphy S.A., Bierman K.L. (2004). A conceptual framework for adaptive preventive interventions. Prev. Sci..

[42] Welch-Brewer C. (2018). Risk-need profiles of serious and chronic female justice-involved youth: Implications for female juvenile correctional programming. Int. J. Offender Ther. Comp. Criminol..

[43] Andrews D.A., Bonta J. (2006). The Psychology of Criminal Conduct.

[44] Gendreau P., Smith P., French S.A., Cullen F.T., Wright J.P., Blevins K.R. (2006). The theory of effective correctional intervention: Empirical status and future directions. Advances in Criminological Theory, Taking Stock: The Status of Criminological Theory.

[45] Liberati A., Altman D.G., Tetzlaff J., Mulrow C., Gøtzsche P.C., Ioannidis J.P.A., Moher D. (2009). The PRISMA statement for reporting systematic reviews and meta-analyses of studies that evaluate health care interventions: Explanation and elaboration. PLoS Med..

[46] Thyer B.A. (2010). The Handbook of Social Work Research Methods.

[47] Roberts-Lewis A.C., Welch-Brewer C.L., Jackson M.S., Pharr O.M., Parker S. (2010). Female justice-involved youth with heart: Preliminary findings of an intervention model for female justice-involved youth with substance use problems. J. Drug Issues.

[48] Harold G.T., Kerr D.C., Van Ryzin M., Degarmo D.S., Rhoades K.A., Leve L.D. (2013). Depressive symptom trajectories among girls in the juvenile justice system: 24-month outcomes of an RCT of multidimensional treatment foster care. Prev. Sci..

[49] Banks B., Kuhn T., Blackford J.U. (2015). Modifying dialectical behavior therapy for incarcerated female youth: A pilot study. J. Juv. Justice.

[50] Crosby S.D., Somers C.L., Day A.G., Zammit M., Shier J.M., Baroni B.A. (2017). Examining school attachment, social support, and trauma symptomatology among court-involved, female students. J. Child. Fam. Stud..

[51] Linehan M.M. (1993). Skills Training Manual for Treating Borderline Personality Disorder.

[52] Turchik J., Karpenkov V.V., Ogles B.M. (2007). Further evidence of the utility and validity of a measure of outcome for children and adolescents. J. Emot. Behav. Disord..

[53] Somers C.L., Gizzi T.J. (2001). Predicting adolescents’ risky behaviors: The influence of future orientation, school involvement, and school attachment. Adolesc. Fam. Health.

[54] Matthews B., Hubbard D.J. (2008). Moving ahead: Five essential elements for working effectively with girls. J. Crim. Justice.

[55] Goldstein N.E., Arnold D.H., Weil J., Mesiarik C.M., Peuschold D., Grisso T., Osman D. (2003). Comorbid symptom patterns in female juvenile offenders. Int. J. Law Psychiatry.

[56] Messina N., Grella C.E., Cartier J., Torres S. (2010). A randomized experimental study of gender-responsive substance abuse treatment for women in prison. J. Subst. Abus. Treat..

[57] Bruce E., Waelde L.C. (2008). Relationships of ethnicity, ethnic identity, and trauma symptoms to delinquency. J. Loss Trauma.

[58] Day D.M., Hart T.A., Wanklyn S.G., McCay E., Macpherson A., Burnier N. (2013). Potential mediators between child abuse and both violence and victimization in justice-involved youth. Psychol. Serv..

[59] Lawrence R., Hesse M. (2010). Juvenile Justice: The essentials.

[60] Griffin G., Germain E.J., Wilkerson R.G. (2012). Using a trauma-informed approach in juvenile justice institutions. J. Child. Adolesc. Trauma.

[61] Belknap J., Dunn M., Holsinger K. (1997). Moving Toward Juvenile Justice and Youth Serving Systems that Address the Distinct Experience of the Adolescent Female.

[62] Valentine Foundation (1990). A Conversation About Girls.

[63] Belknap J., Holsinger K., Sapling R.T. (1998). An overview of delinquent girls: How theory and practice have failed and the need for innovative changes. Female Offenders: Critical Perspectives and Effective Interventions.

[64] Mulvany E.P. Highlights from pathways to desistance: A longitudinal study of serious adolescent offenders. Center on Juvenile and Criminal Justice.

[65] Wade D., Varker T., Kartal D., Hetrick S., O’Donnell M., Forbes D. (2016). Gender difference in outcomes following trauma-focused interventions for posttraumatic stress disorder: Systematic review and meta-analysis. Psychol. Trauma.

